# Cyclophosphamide versus mycophenolate mofetil in scleroderma interstitial lung disease (SSc-ILD) as induction therapy: a single-centre, retrospective analysis

**DOI:** 10.1186/s13075-016-1015-0

**Published:** 2016-06-02

**Authors:** Padmanabha D. Shenoy, Manish Bavaliya, Sujith Sashidharan, Kaveri Nalianda, Sreelakshmi Sreenath

**Affiliations:** Centre For Arthritis & Rheumatism Excellence (CARE), NH-47, Nettoor, Kochi, Kerala 682040 India; Department of Rheumatology, Amrita Institute of Medical Sciences, Ponekkara, Kochi, Kerala India; Department of Medicine, Amrita Institute of Medical Sciences, Ponekkara, Kochi, Kerala India

**Keywords:** Scleroderma interstitial lung disease, Cyclophosphamide, Mycophenolate mofetil

## Abstract

**Background:**

Scleroderma is a systemic autoimmune disease characterized mainly by skin manifestations and involvement of various visceral organs, especially the lungs. Lung involvement is the leading cause of mortality in patients with scleroderma. There are data to suggest that cyclophosphamide (CYC) and mycophenolate mofetil (MMF) are effective in the management of scleroderma interstitial lung disease (SSc-ILD) but no head to head comparative data are available to date.

**Methods:**

For the last 3 years, patients with SSc-ILD have been treated at our centre by protocol-based administration of intravenous CYC and MMF. Results of lung function tests (spirometry) were recorded at baseline, 3 months and 6 months in every patient. The clinical records of patients with systemic sclerosis and significant ILD, who were not previously exposed to any immunosuppressant and were treated with MMF OR CYC, were reviewed. The efficacy of treatment was assessed by the change in forced vital capacity on spirometry.

**Results:**

Of the total 57 patients included in the analysis, 34 were treated with MMF and 23 were treated with CYC. Mean duration of illness was 4.19 ± 2.82 years in the MMF and 6.04 ± 5.96 years in the CYC group. After 6 months of therapy, FVC increased by 10.84 ± 13.81 % in the CYC group and by 6.07 ± 11.92 % in the MMF group. This improvement from baseline was statistically significant in both groups (*P* < 0.01). The improvement was comparable with no statistically significant differences between groups (*P* = 0.373). There were no major adverse events reported in either arm.

**Conclusion:**

Both MMF and CYC were equally effective in stabilizing lung function in patients with scleroderma and ILD.

## Background

Lung diseases in scleroderma (SSc) are the leading disease-related cause of mortality, most typically including interstitial lung disease (ILD) and/or pulmonary arterial hypertension (PAH) [1]. Approximately 80 % of patients have evidence of pulmonary fibrosis at postmortem examination [2] or on high-resolution computed tomography (HRCT), although clinically evident disease is present only in approximately 40 % of patients [3]. The 10-year survival rates of patients with SSc in an Italian study involving 915 patients was 64.9 % in those with lung involvement in comparison to 80.6 % in those without lung involvement [4].

For more than 15 years, cyclophosphamide (CYC) is used in the treatment of SSc-ILD. CYC is a cytotoxic immunosuppressive agent that suppresses lymphokine production and modulates lymphocyte function by alkylating various cellular constituents and depressing the inflammatory response via normalization of neutrophilia and healing of vascular endothelial cells [5]. CYC is the only agent shown to at least stabilize lung function in a randomized, controlled trial [6].

Mycophenolic acid, the active metabolite of mycophenolate mofetil (MMF) and mycophenolate sodium (MS), inhibits inosine monophosphate dehydrogenase and depletes guanosine nucleotides. It is thus thought to decrease the activity of inflammatory cells and to produce anti-fibrotic and immunomodulatory effects. Mycophenolic acid has emerged recently as a promising therapy for SSc [7]. Evidence supporting its use derives from small, uncontrolled, single-arm studies, suggesting that mycophenolate is a safe therapeutic modality associated with functional stabilization of SSc-ILD [8–15].

No head to head data comparing the efficacy of CYC and MMF are available to date. The objective of the study was to compare the efficacy of MMF and CYC in patients with SSc-ILD for improvement in lung function.

## Methods

### Study design

#### A single-centre, retrospective study

##### Patients and treatment regimes

At our centre MMF and CYC were used, in a protocol-based manner, for the treatment of SSc-ILD. MMF was initiated with 500 mg once a day and was increased to maximum tolerable dose or to a maximum dose of 3 g daily. Patients were advised to take MMF 30 minutes before food or 2 h after food. The starting dose of CYC was 600 mg/m^2^ given as an intravenous (IV) infusion. Six monthly pulses of CYC were given and the dose was increased to 1.2 g as tolerated. Adequate hydration was maintained. Selection of the drug for induction therapy was based on the patient’s willingness to receive a particular drug after explaining the side effects and treatment costs of each of the drugs in detail to the patient. After detailed baseline evaluations every patient underwent a monthly clinical examination. Forced vital capacity (FVC) was assessed by spirometry at baseline, 3 and 6 months.

We carried out a retrospective analysis of clinical records of the patients attending the rheumatology outpatient department (OPD), who had ILD and had undergone treatment with intravenous pulses of CYC or MMF in the last 3 years. Patients were included for analysis if they were over 18 years of age, fulfilled the American College of Rheumatology (ACR) 2013 classification criteria for SSc with significant ILD as defined by FVC ≤80, and had evidence of ILD on thoracic HRCT. Patients were excluded if they had end-stage lung disease and FVC <20 % and were oxygen dependent, clinically significant abnormalities on HRCT that were not attributable to SSc, clinically significant cytopoenia (Hb <10 g %, leucocyte count <4000 per cubic millimeter, platelet count <1,50000 per cubic millimeter), altered liver function on tests (liver enzymes >2 times normal and/or bilirubin >1.5 times the upper limit of normal) or altered renal function (unexplained haematuria or serum creatinine >2.0 mg/dl), or who had already been treated with CYC or MMF or other immunosuppressants such as cyclosporin or rituximab, or had severe cardiac disease, with moderate to severe PAH (right ventricle systolic pressure (RVSP) >50 mm Hg) measured by transthoracic echocardiography (TTE), or were chronic smokers, or were pregnant or lactating. Those patients who were unable to perform spirometry or whose data were missing were not included in the study.

The study was performed in accordance with the Declaration of Helsinki and received approval from the institutional ethics committee of Amrita institute of medical science and research institute. Informed consent was not relevant as it was a retrospective study.

#### Lung function test

Spirometry (MicroQuark-Cosmed PFT suit) was performed by a highly trained and experienced technician in all the patients so as to minimize interobserver variability to achieve a high degree of reproducibility (interclass correlation coefficient of 0.96). The results were expressed as percentage of predicted for each patient. Depending on the FVC values, the severity of ILD was classified as mild (70–80 %), moderate (50–69 %) and severe (<50 % predicted values) according to Medsger’s severity scale [16]. In order to evaluate the response to treatment by means of spirometry values, the recommendations of the American Thoracic Society (ATS) [17] were followed. According to these recommendations, improvement is defined by an increase in FVC ≥10 %, stabilization by change in FVC <10 % and worsening by a reduction in FVC ≥10 %. Spirometry was carried out in all patients at baseline (before treatment was started) and repeated at 3 and 6 months. Baseline and 6-month data were used for comparisons.

#### Statistical analysis

Data were presented as mean ± standard deviation for numerical variables. Continuous variables were analysed within groups using the paired *t* test, and the independent two-sample *t* test or the Mann–Whitney test (non-parametric data) was used to compare variables in the two groups. The chi square test was used to test the associations between categorical variables. Differences were considered significant at *P* ≤ 0.05. Data were analysed using IBM SPSS Version 20 software.

## Results

As summarized in Table 1, both groups were comparable in terms of demographic data and other baseline variables. There were 23 patients with SSC-ILD (18 female and 5 male) in the CYC group and 34 patients (31 female and 3 male) in the MMF group. The mean age was around 46 years in both groups. The mean disease duration, defined as the time from initiation of the first non- Raynaud’s symptom, was around 5 years. During the study period 17 patients in the CYC and 30 patients in the MMF group were on low-dose steroids (≤5 mg/day). The average cumulative dose of CYC was 7.2 g and the average maximum dose of MMF was 2.62 g/day.

**Table 1:** Demographic and baseline clinical characteristics of patients in the cyclophosphamide (CYC) and mycophenolate mofetil (MMF) groups

Characteristics	CYC (n = 23)	MMF (n = 34)	*P* value for significance
Age, mean ± SD	46 ± 10.34	45.24 ± 13.87	0.82
Female, *n* (%)	18 (78.2)	31 (91.18)	0.24
Disease duration in years, mean ± SD	6.04 ± 5.96	4.19 ± 2.82	0.11
Clinical features, *n* (%)			
Breathlessness	21 (91.3)	30 (88.2)	0.53
Arthritis	23 (100)	28 (82.4)	0.07
Skin changes	21 (91.3)	30 (88.2)	0.53
Raynaud’s symptoms	17 (74)	18 (52.9)	0.16
Baseline FVC %, mean ± SD	48.74 ± 15.67	53.44 ± 13.69	0.23
Pulmonary artery hypertension, *n* (%)	2 (2.7)	4 (11.8)	0.63
Anti-scl-70-positive, *n* (%)	12 (52.17)	16 (47.18)	0.41

*FVC* forced vital capacity

Both the groups had similar lung function at baseline as evidenced by a baseline FVC of 48.74 ± 15.67 % in the CYC group and 53.44 ± 13.69 % in the MMF group. PAH was detected in two patients in the CYC group and four patients in the MMF group.

**Fig. 1:**
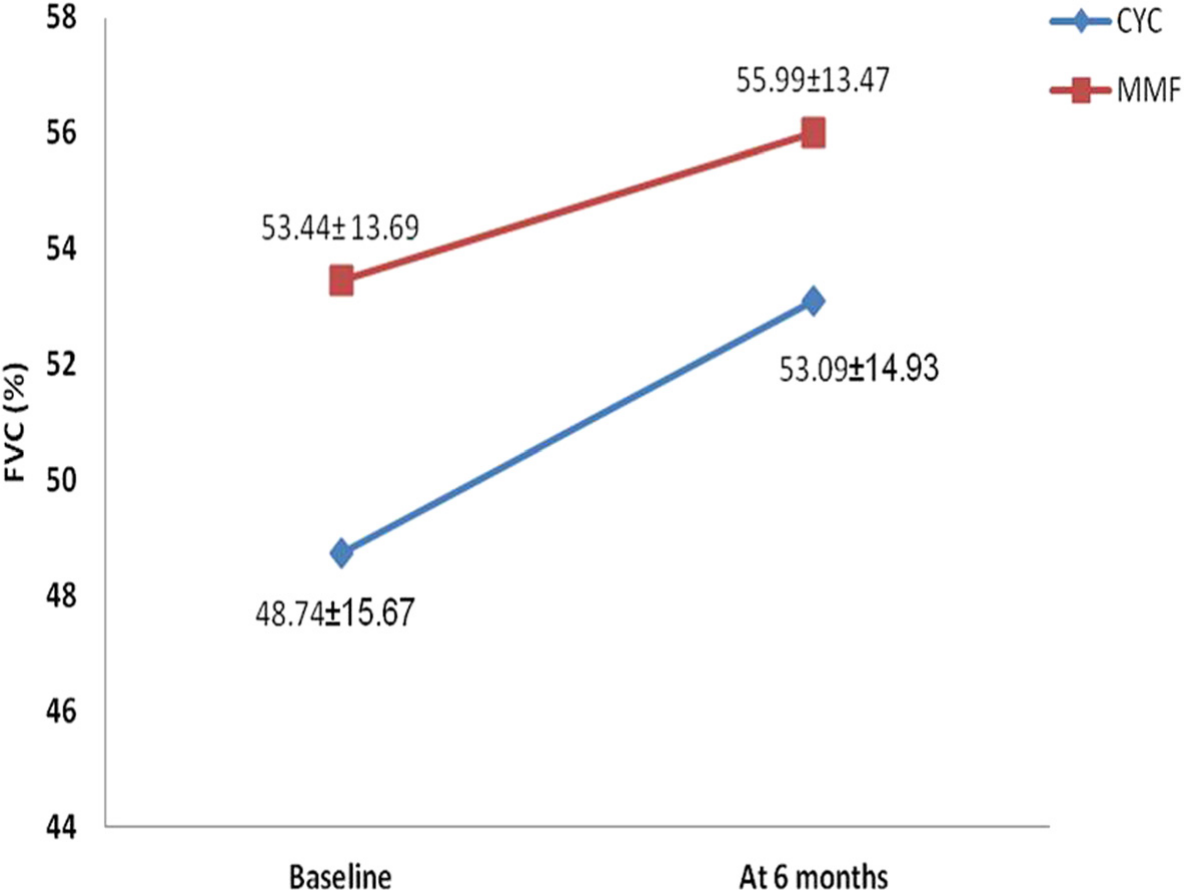
Mean change in percentage forced vital capacity (*FVC*) at baseline and 6 months in the mycophenolate mofetil (*MMF*) and cyclophosphamide (*CYC*) groups

**Fig. 2:**
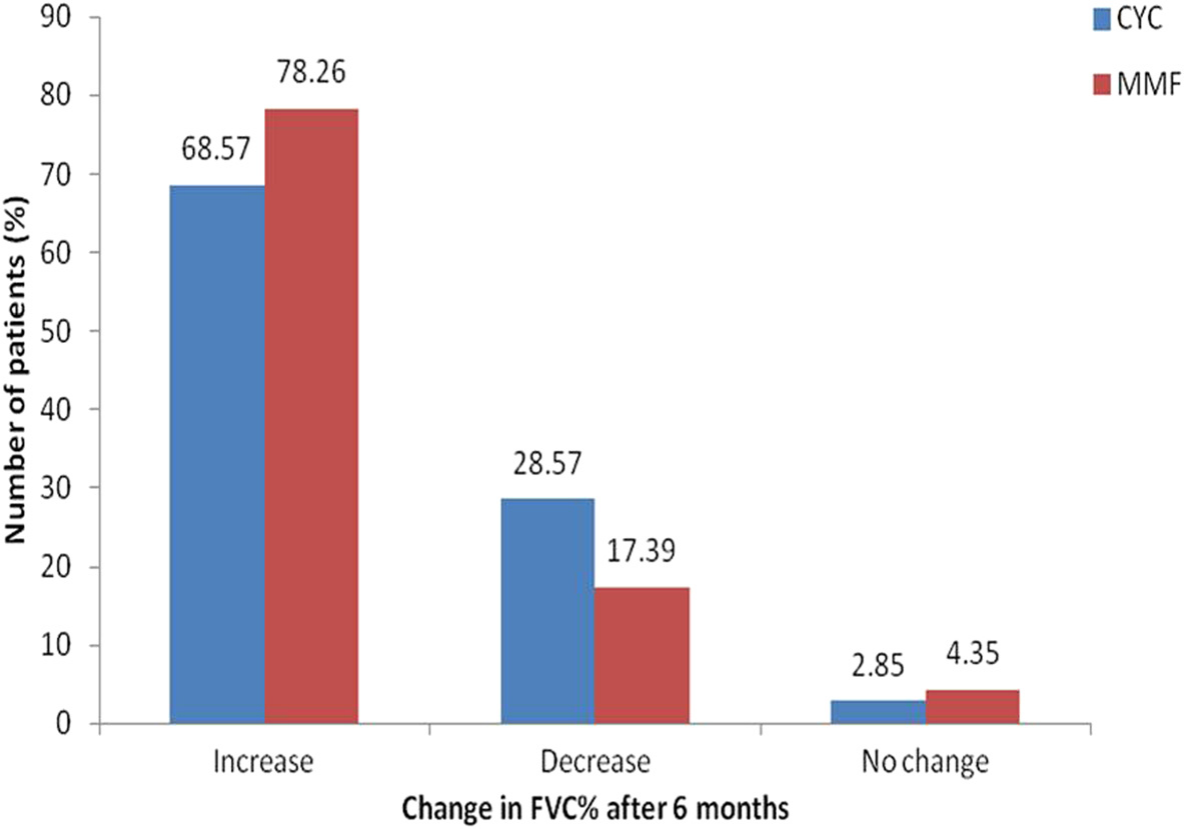
Change from baseline in percentage forced vital capacity (*FVC%*) at 6 months in the mycophenolate mofetil (*MMF*) and cyclophosphamide (*CYC*) groups: 68.57 %, 28.57 % and 2.85 % of patients in the CYC group had increased, decreased and unchanged FVC% respectively, and 78.26 %, 17.39 % and 4.35 % of patients in the MMF group had increased, decreased and unchanged FVC% after 6 months of treatment

As shown in Fig. 1, the mean FVC increased to 55.99 ± 13.47 % from the baseline value of 53.44 ± 13.69 % in the MMF group (*P* = 0.003). Similarly, mean FVC improved to 53.09 ± 14.93 % from the baseline value of 48.74 ± 15.67 % (*P* = 0.003) in the CYC group. The mean percentage increase in FVC at 6 months compared to baseline was 10.84 ± 13.81 % in the CYC group and 6.07 ± 11.92 % in the MMF group. Both improvements were statistically significant when compared to baseline. Hence, as per the ATS recommendation, the CYC group had an improvement, as the percentage increase in FVC was >10 %, but FVC was only stabilized in the MMF group (FVC change <10 %). When the change in FVC at 6 months was compared in the two groups, there was no significant difference (*P* = 0.232). As shown in Fig. 2, when numerical values were considered, 68.57 %, 28.57 % and 2.85 % of patients in the CYC group had increased, decreased and unchanged percentage FVC, respectively, and 78.26 %, 17.39 % and 4.35 % of patients in the MMF group had increased, decreased and unchanged percentage FVC after 6 months of treatment.

Both drugs were generally well tolerated in all patients except for nine patients in the CYC group and four patients in the MMF group who developed lower respiratory tract infection; this was treated with a short course of antibiotics. No exacerbation of ILD, hospital admissions, or fatalities occurred in either group during treatment.

## Discussion

The results of this retrospective analysis demonstrate that both CYC and MMF were equally effective in preserving lung function in patients with SSc-ILD. Prevention of disease progression is considered the most important aim in SSc-ILD [18].

In a landmark study, The Scleroderma Lung Study Research Group noted that oral CYC achieved a modest but significant beneficial effect on lung function and patients’ quality of life, but unfortunately, it also provoked a serious adverse event in six patients [6]. After the first year these patients were observed without any immunosuppression and by 2 years the improvement in lung function that had been observed in the CYC arm was lost [19]. The modest and transitory benefit of CYC in patients with SSc-ILD and the substantial toxicity of the drug compels us to search for safer alternatives, especially for younger patients with mild disease, who would benefit from longitudinal administration of therapeutic modalities with minimal side effects, and to reserve more aggressive and potentially more beneficial cytotoxic regimens for later stages of the disease course.

To date, all the available information on the use of mycophenolate in the treatment of SSc-related ILD comes from small-scale, single-arm and open-label studies. A recent meta-analysis, which includes six studies and a total of 69 patients, the majority of whom had diffuse cutaneous SSc-related ILD, confirmed the acceptable safety and tolerability profile of mycophenolate. In the primary analysis, four out of six studies reported statistically significant functional improvement following 12-month oral administration of MMF or MS, and the remaining two studies reported stabilization of disease; however, a pooled analysis did not corroborate this finding. In particular, in the overall analysis of 69 patients, MMF or MS treatment was not associated with a statistically significant beneficial functional effect as assessed by both FVC and the diffusing capacity of the lungs for carbon monoxide (DLCO) [20]. A more recent, study by Fischer et al. [9], including 125 patients with connective tissue disease-associated ILD (of whom 44 patients had SSc-ILD) treated with mycophenolate for a median of 897 days, showed stabilization or improvement of lung function.

The results of the current study show both MMF and CYC were not only able to stabilize lung function but also there was a numerical increase in FVC in the majority of patients. In view of the expected annual decline in lung function over time among patients with SSc-ILD [21], it is reasonable to state that a marginal increase in FVC or even a stabilization of functional status through the disease course is a great achievement, especially when the therapeutic agent used presents with an excellent safety profile tested longitudinally.

Besides this, it is important to note that in our study the enrolled patients had a mean disease duration of around 5 years with moderate to severe ILD prior to administration of CYC or MMF. It can be argued that stabilization can happen in a group of patients with SSc and the results of the study merely reflect the natural course of disease. But to prove this point we need a placebo arm, which appears to be unethical in a deadly disease like SSc-ILD. Moreover, improvement in lung function is not part of the natural course of the disease and the patients with FVC <70 % are more likely to deteriorate than others. Scleroderma lung study 2 (SLS2), the results of which are currently available in abstract form, compared oral cyclophosphamide with MMF. The results showed that the improvement in FVC is comparable in both the arms at the end of 2 years. These results are in concurrence with ours, although in SLS 2 oral CYC was used instead of IV CYC.

Our study is limited by the lack of a control arm for those who were not treated, randomization and long follow up. The 6-minute walking test was not used as an outcome measure as it has been proven that features like pain and musculoskeletal involvement can influence the result [22], and HRCT could not be performed in all patients at 6 months due to financial constraints. As the reproducibility of FVC is better than that of DLCO and DLCO is less specific than FVC for lung function, DLCO was not chosen as an outcome measure.

## Conclusion

We support the use of immunosuppressants in patients with scleroderma ILD. Although MMF and CYC may be equally effective, MMF may be preferred due to the long-term toxicity of CYC. Larger randomized controlled studies are sorely needed to support this premise.

## Abbreviations

ACR: American college of rheumatology
ATS: American Thoracic Society
CYC: cyclophosphamide
DLCO: diffusing capacity of the lungs for carbon monoxide
FVC: forced vital capacity
HRCT: high-resolution computed tomography
ILD: interstitial lung disease
IV: intravenous
MMF: mycophenolate mofetil
MS: mycophenolate sodium
PAH: pulmonary arterial hypertension
RVSP: right ventricle systolic pressure
SLS2: Scleroderma lung study 2
SSc: scleroderma
SSc-ILD: scleroderma interstitial lung disease
TTE: transthoracic echocardiography
